# Starvation induced autophagy promotes the progression of bladder cancer by LDHA mediated metabolic reprogramming

**DOI:** 10.1186/s12935-021-02303-1

**Published:** 2021-11-07

**Authors:** Tinghao Li, Hang Tong, Hubin Yin, Yi Luo, Junlong Zhu, Zijia Qin, Siwen Yin, Weiyang He

**Affiliations:** 1 The First Affiliated Hospital of Chongqing Medical University, Chongqing, 400016 China; 2 Central Laboratory, The First Affiliated Hospital of Chongqing Medical University, Chongqing, 400016 China; 3 Department of Urology, The First Affiliated Hospital of Chongqing Medical University, 1 Youyi Road, Yuzhong District, Chongqing, 400016 People’s Republic of China

**Keywords:** Bladder cancer, Autophagy, Glycolysis, LDHA, Wnt/β-catenin signalling, Axin1

## Abstract

**Background:**

Aberrant autophagy and preternatural elevated glycolysis are prevalent in bladder cancer (BLCA) and are both related to malignant progression. However, the regulatory relationship between autophagy and glycolytic metabolism remains largely unknown. We imitated starvation conditions in the tumour microenvironment and found significantly increased levels of autophagy and aerobic glycolysis, which both regulated the progression of BLCA cells. We further explored the regulatory relationships and mechanisms between them.

**Methods:**

We used immunoblotting, immunofluorescence and transmission electron microscopy to detect autophagy levels in BLCA cells under different treatments. Lactate and glucose concentration detection demonstrated changes in glycolysis. The expression of lactate dehydrogenase A (LDHA) was detected at the transcriptional and translational levels and was also silenced by small interfering RNA, and the effects on malignant progression were further tested. The underlying mechanisms of signalling pathways were evaluated by western blot, immunofluorescence and immunoprecipitation assays.

**Results:**

Starvation induced autophagy, regulated glycolysis by upregulating the expression of LDHA and caused progressive changes in BLCA cells. Mechanistically, after starvation, the ubiquitination modification of Axin1 increased, and Axin1 combined with P62 was further degraded by the autophagy–lysosome pathway. Liberated β-catenin nuclear translocation increased, binding with LEF1/TCF4 and promoting LDHA transcriptional expression. Additionally, high expression of LDHA was observed in cancer tissues and was positively related to progression.

**Conclusion:**

Our study demonstrated that starvation-induced autophagy modulates glucose metabolic reprogramming by enhancing Axin1 degradation and β-catenin nuclear translocation in BLCA, which promotes the transcriptional expression of LDHA and further malignant progression.

**Supplementary Information:**

The online version contains supplementary material available at 10.1186/s12935-021-02303-1.

## Introduction

Bladder cancer (BLCA) is the second most common genitourinary tumour, with over 430,000 patients diagnosed worldwide per year [1]. Various kinds of solid cancer cells, such as BLCA, always live in a tumour microenvironment (TME) with intermittent starvation, hypoxia and inflammatory microenvironments due to excessive multiplication and lack of vascular perfusion [2, 3]. Cancer cells’ unlimited proliferation and hyperadaptation to drastic changes are generally maintained by adaptive metabolic reprogramming caused by harsh living conditions, accelerating the development of cancer cells [4–6]. The conflict between nutrient deprivation and the promotion of cancer cell malignancy has attracted great attention. Thus, the effects of TME on BLCA cells remain to be addressed.

Autophagy acts as a cancer suppressor in normal tissues, while sometimes it is overactivated to counteract different kinds of adverse conditions in cancer cells [7]. Autophagy may play a protective role in cancer cells under conditions of intermittent starvation or hypoxia [8, 9]. The autophagic process can be explained in two steps. First, autophagosomes degrade the ribosomal region of the rough endoplasmic reticulum, breaking down cargos such as redundant proteins and organelles. Then, autophagosomes fuse with lysosomes to form autophagic lysosomes for further degradation. This facilitates the cycling and reutilization of materials and energies [10–12]. The effects of a hostile TME on autophagy and the influence of elevated autophagy on cancer cells remain unknown. Epigenetic modification of metabolic reprogramming is one such effect. It is known that cells require three main nutrients for survival, and under nutrient deprivation, cancer cells can reprogram their metabolic pathways. This is sometimes triggered by enhanced autophagy and leads to malignant progression [13, 14].

Glycolysis is a characteristic feature of tumour cells and is termed the Warburg effect. The mechanisms of glycolysis and key enzymes and regulators of glycolytic pathways have recently drawn wide attention. It was reported that the regulation of key enzymes in glycolysis at the transcriptional or translational level affects both the transformation of glucose metabolism and the progressive transformation of cancer cells. Starvation could promote the translational expression of monocarboxylate transporter 1 by upregulating autophagy and accelerating metastasis in hepatic cancer [15]. Phosphofructokinase and platelets (PFKP) are also known to have a core role in the malignant process in oral squamous cell carcinoma [16]. This study aimed to investigate the connections between aberrant autophagy and enhanced aerobic glycolysis in BLCA.

Our current study aimed to explore changes in BLCA cells under starvation and detail the mechanisms. The findings of this study revealed that transient starvation could enhance autophagic flux. This is achieved by accelerating the ubiquitin-mediated degradation of Axin1 and liberating the transcriptional expression of the key enzyme lactate dehydrogenase A (LDHA) to modulate the levels of glycolysis, allowing the malignant progression of BLCA.

## Materials and methods

### Cell culture and treatment

Two strains of BLCA cells (T24 and UM-UC-3) were obtained from American Type Culture Collection (Manassas, VA). Normally treated groups were grown in Roswell Park Memorial Institute (RPMI) 1640 medium for T24 cells or Dulbecco’s modified Eagle’s medium (DMEM) for UM-UC-3 cells supplemented with 10% foetal bovine serum (Gibco, Thermo Fisher Scientific, MA). Starvation-incubated groups were treated with Hank’s balanced salt solution (HBSS; Boster Biotechnology, Wuhan, China) for six hours and were then recovered in complete medium for further experiments [17]. Chloroquine (CQ, 20 mM; Sigma-Aldrich, USA), 3‐methyladenine (3-MA, 5 mM; Selleck Chemicals, Houston, TX), 2-deoxy-D-glucose (2-DG, 5 mM; Selleck Chemicals), MG132 (50 mM; Selleck Chemicals) and PNU-74654 (50 mM; Selleck Chemicals) were used to treat cell lines with different inhibitors.

### Small interfering RNA interference assay

All small interfering RNAs (siRNAs) targeting human LDHA or a negative control were designed and synthesized by GenePharma (Shanghai, China) and transfected into different cell lines with Lipofectamine 2000 (Invitrogen, Carlsbad, CA) according to the manufacturer’s instructions. The sequences are listed in Table 1.

**Table 1:** Sequences of small interfering RNA or Primer sequences for quantitative real‐time PCR

Designation	Genes	Sequences (5′–3′)	Organism
Primer	G6PD	F: -AACATCGCCTGCGTTATCC-	Homo sapiens
		R:- TGACCTTCTCATCACGGACG-	
	HK2	F: -GCCCGCCAGAAGACATTAG-	
		R: -TGCTCAGACCTCGCTCCAT-	
	PDK1	F: -GAGGGTTACGGGACAGATGC-	
		R: -GCCTCGTGGTTGGTGTTGT-	
	PKM	F: -GCTGTGGACTTGCCTGCTGT-	
		R: -GCCTTGCGGATGAATGACG-	
	GLUT1	F: -GTATGTGGAGCAACTGTGTGGT-	
		R: -CTCGGGTGTCTTGTCACTTTG-	
	LDHA	F: -ATTAAGCTGTCATGGGTGGGTC-	
		R: -CAGAGAGACACCAGCAACATTCA-	
	PFKP	F: -TGTATTCAGAAGAGGGCAAAGG-	
		R: -AGTTTCTATCAAATGGAGAGGGTG-	
	CTNNB1	F:- CATGCACCTTTGCGTGAGCA-	
		R:- CCCCCTCCACAAATTGCTGC-	
	β-actin	F: -AGAAAATCTGGCACCACACCT-	
		R: -GATAGACAGCCTGGATAGCA-	
si-RNA	LDHA-siRNA-1	F:- GGAGAAAGCCGUCUUAAUUTT -	Homo sapiens
		R:- AAUUAAGACGGCUUUCUCCTT -	
	LDHA-siRNA-2	F:- GACUGAUAAAGAUAAGGAATT -	
		R:- UUCCUUAUCUUUAUCAGUCTT -	
		F:- GAUUAAGGGUCUUUACGGATT -	
	LDHA-siRNA-3	R:- UCCGUAAAGACCCUUAAUCTT -	

### Quantitative real‐time polymerase chain reaction

Total RNA was extracted from T24 or UM-UC-3 cells processed under different experimental conditions. A PrimeScript RT reagent kit (TaKaRa, Osaka, Japan) was used to reverse‐transcribe RNA (1 mg) to cDNA. Real-time qPCR was performed with SYBR Green (TaKaRa) on an ABI 7500 Real‐Time PCR System (Applied Biosystems), and the entire process followed the manufacturer’s instructions. Data were standardized to β‐actin using the 2^−ΔΔCt^ method. The primer sequences are listed in Table 1.

### Immunoblots

Total protein was extracted using radioimmunoprecipitation assay lysis buffer (Beyotime, China). An NE-PER™ Nuclear Cytoplasmic Extraction Reagent kit (Thermo Fisher Scientific, MA) was used according to the manufacturer’s instructions for extracting nuclear and cytoplasmic proteins from the different groups.

A 12% sodium dodecyl sulfate‐polyacrylamide gel was chosen for total protein separation, and the proteins were then transferred to nitrocellulose membranes (Millipore, USA). The membranes were incubated with primary antibodies, including anti-LC3B (Abcam Cat# ab192890, RRID: AB_2827794), anti-P62/SQSTM1 (Abcam Cat# ab207305, RRID: AB_2885112), anti-β-actin (Proteintech# 20536-1-AP), anti-LDHA (Cell Signaling Technology Cat# 3582, RRID: AB_2066887), anti-β-catenin (Cell Signaling Technology Cat# 8480), anti-p-β-catenin (Cell Signaling Technology Cat# 4176), anti-c-Myc (Covance Cat# MMS-150P-1000, RRID: AB_291322), anti-GSK3-β (Cell Signaling Technology Cat# 121456), anti-Axin1 (Cell Signaling Technology Cat# 2087, RRID: AB_2274550) and anti-Histone H3 (Cell Signaling Technology Cat# 4499, RRID: AB_10544537). Enhanced chemiluminescence reagents (Millipore, USA) were used to assess protein expression.

### Cell proliferation and viability

Cell proliferation was demonstrated by Cell Counting Kit-8 (CCK-8, Boster Biotechnology) and colony formation assays. For the CCK-8 assay, cells processed under different experimental conditions were inoculated onto 96-well plates at an initial density of 5000 cells per well. CCK-8 reagent (10 µl) was added to each well at different times, and the normal incubation or NC transfection group was chosen as the negative control. For the colony formation assay, cells were inoculated onto 6- and 24-well plates with complete medium for 10 days at a density of 500 cells per well when inoculated into 6-well plates at a density of 100 cells per well in 24-well plates. Colonies were fixed with 4% paraformaldehyde fixation solution (P0099, Beyotime, China) and stained with 0.1% crystal violet staining solution (C0121, Beyotime).

Cell activity, used to reflect the sensitivity of cells to chemotherapy, was also evaluated by CCK-8 assay. In accordance with the methods and procedures mentioned above, different groups were inoculated onto 96-well plates and treated with cisplatin at the indicated concentrations (0, 0.2, 0.5, 1, 2, 5, 10 and 20 µM) for an additional 48 h of incubation.

### Transwell migration and invasion assays

Migration and invasion assays were performed using a 24-well Transwell chamber. For the migration assay, cells were inoculated in the upper chamber. Migrated cells that attached to the substratum of the membrane were observed and photographed after fixation and staining. To evaluate the migration abilities of different groups, migrated cells in each chamber were counted in five isolated fields under a microscope at 200× magnification. For the invasion assay, 50 ml of Matrigel mixture was added to the upper chamber in advance. The remaining procedures and methods were the same as above.

### Lactate production and glucose consumption measurements

Lactate and glucose concentrations in the culture supernatants were detected using a lactate assay kit (Solarbio, Beijing, China) and a glucose assay kit (Solarbio), respectively, according to the manufacturer’s instructions, and absorbance values were measured at the corresponding absorbance. The results were normalized by the number of cells in each sample in the culture plates, and lactate production and glucose consumption were calculated by comparison with normal RPMI 1640 medium or DMEM, respectively.

### Transmission electron microscopy

After different treatments, the cells were fixed in 3% glutaraldehyde electron microscope fixation solution, gradually dehydrated in a 50–100% ethanol gradient and embedded in araldite. Samples were chopped into ultrathin sections and stained with uranyl acetate and lead citrate. Images were obtained by transmission electron microscopy.

### Immunofluorescence

Different groups of cells were fixed for 30 min. After permeabilization with 1% Triton X-100, primary antibodies, including anti-LC3B (Abcam Cat# ab192890, RRID: AB_2827794) or anti-β-catenin (Cell Signaling Technology Cat# 8480), were added. The secondary antibody Alexa 488-conjugated goat anti-rabbit IgG (Beyotime Cat# A0423, RRID: AB_2891323) was then added. After counterstaining with DAPI (Beyotime Cat# C1002), cells were observed under a confocal microscope.

### Immunoprecipitation (IP) and ubiquitination assay

Cells were harvested and lysed by IP lysis buffer (Beyotime Cat# P0013). Samples were centrifuged, supernatants were collected, and 2.5 mg of anti-Axin1 (Cell Signaling Technology Cat# 2087, RRID: AB_2274550), anti-p62 (Abcam Cat# ab207305, RRID: AB_2885112) or anti-IgG (Cell Signaling Technology Cat# 3900S) antibodies were added. Protein A/G immunoprecipitation beads were added to the suspension. Bead-binding proteins were isolated by a magnetic grate. IgG was used as the negative control. To detect and inhibit ubiquitin protein, 20 mM anti-ubiquitin (Cell Signaling Technology Cat# 3933, RRID: AB_2180538) and MG132 (Selleck Chemicals Cat# S2619) dissolved in DMSO were also selected and applied.

### Animal experiments

BALB/c nude mice (HFKBIO, Beijing, China) were fed under standard conditions. A total of 5 × 10^6^ cells with normal or starvation treatment were subcutaneously inoculated. Measurements were taken every 3 days on the tumour volume, which was calculated as follows: tumour volume (mm^3^) = 0.5 × longest diameter × shortest diameter^2^. When the average tumour volume reached 30 mm^3^, an equal amount of CQ (50 mg/ml dissolved in 0.9% saline, Sigma-Aldrich) or 0.9% saline was then administered intraperitoneally every 3 days. Two weeks later, the mice were euthanized with a high concentration of carbon dioxide after 3% pentobarbital sodium deep anaesthesia, and the size of the tumours was recorded for further analysis.

### Clinical data for human tissue specimens and bioinformatic analysis

Fifteen pairs of cancer tissues and adjacent tissues of patients who underwent radical cystectomy for BLCA were collected at The First Affiliated Hospital of Chongqing Medical University. For bioinformatic analysis, immunohistochemical (IHC) images of the expression of LDHA in normal urothelial and urothelial cancer cells were downloaded from the Human Protein Atlas database.

### Statistical analysis

Experiments were independently repeated, and representative images are shown in the figures. The results of analyses are presented as the mean ± standard deviation. Unpaired Student’s *t* tests were performed to compare the differences between two groups. Data from multiple groups were analysed by the Mann–Whitney test. A P value < 0.05 was considered statistically significant, and significance levels were set to *P < 0.05, **P < 0.01, ***P < 0.001 or nonsignificant (ns).

## Results

### Starvation of the microenvironment enhances the malignant progression of BLCA

**Fig. 1:**
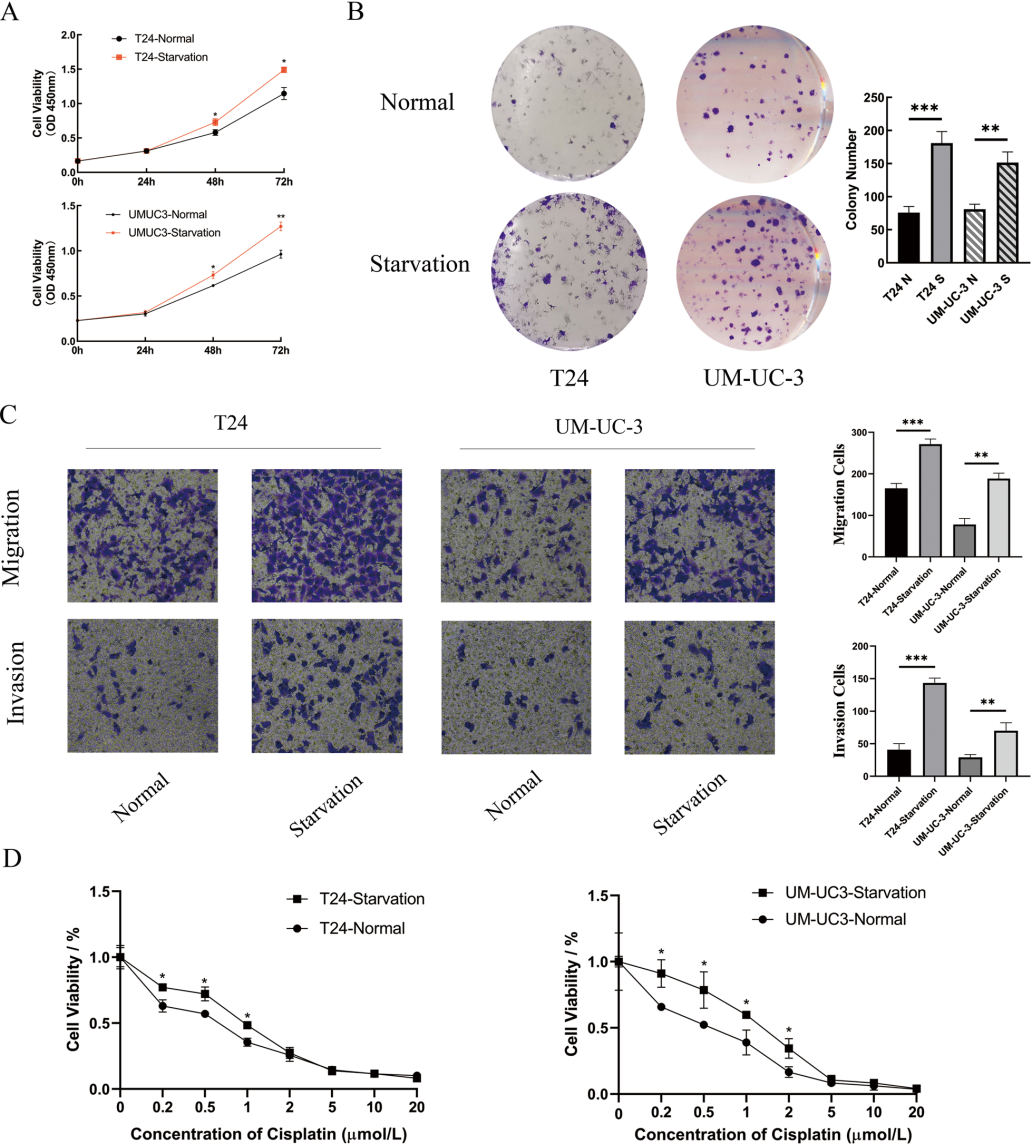
The Microenvironment of Starvation Enhanced the Malignant Progression of BLCA. **a** The viability levels of two strains of BLCA cells after 6 h of normal or HBSS treatment were measured at 0, 24, 48 and 72 h (n = 3, Mann–Whitney test, P* < 0.05). **b** Colony-forming assays demonstrated that the proliferation abilities of T24 and UM-UC-3 cells increased significantly after starvation (n = 3, unpaired Student’s *t* test, P** < 0.01, P*** < 0.001). **c **Cell Transwell migration and invasion assays were chosen to detect the migration and invasion abilities between the normal and starvation groups, and the results indicated that migration and invasion abilities were promoted after starvation. **d** Viabilities of two strains with different treatments were measured after processing with graded concentrations of cisplatin for 48 h (n = 3, nonparametric Mann–Whitney test, P* < 0.05)

Two strains of BLCA cells with high malignancy, T24 and UM-UC-3, were selected for subsequent experiments. Cells were treated with Hank’s buffered salt solution (HBSS) for 6 h to simulate the transient starvation environment and were then recovered [17]. After starvation, proliferation was promoted compared to that of the control group in the CCK-8 assay (Fig. 1A). This finding was also confirmed by the clone formation assay (Fig. 1B). As shown in Fig. 1C, the ability to migrate was enhanced after starvation. Invasion was also promoted significantly relative to that in the normal culture group. Cisplatin was employed in the drug sensitivity test since it is widely applied in the chemotherapy of BLCA patients. With the use of the CCK-8 assay, T24 and UM-UC-3 cells were found to exhibit higher resistance to cisplatin after starvation (Fig. 1D).

### Starvation-induced autophagy promotes the progression of BLCA

**Fig. 2:**
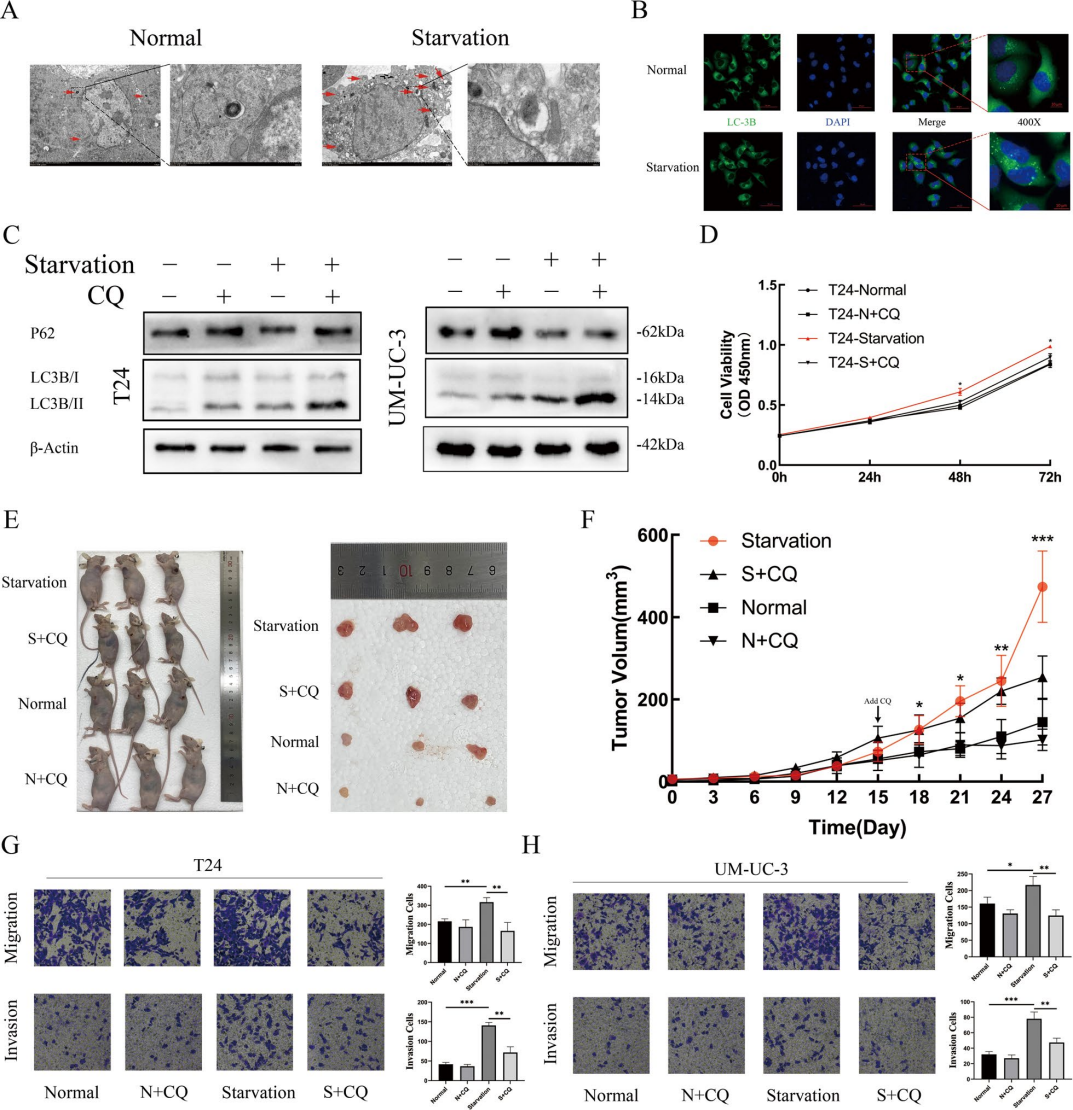
Starvation-Induced Autophagy Promoted the Progression of BLCA. **a **After normal or HBSS treatment, T24 cells were fixed, and the formation of autophagosomes was observed by transmission electron microscopy. Autophagosomes are marked by red arrows (scale bar, 2 mm). **b** Immunofluorescence staining of LC3B was performed in T24 cells with normal or HBSS treatment, and the aggregation of LC3B represented the formation of autophagosomes. **c** Western blotting was used to detect the expression of LC3B-I/II and SQSTM1/P62 to verify the efficiency of autophagic flux inhibition by CQ in different strains of cells and different treatments. **d** CCK-8 assay demonstrated the viabilities of T24 cells after treatment, including normal, normal supplemented with CQ, starvation only and starvation supplemented with CQ (n = 3, Mann–Whitney test, P* < 0.05, P** < 0.01, P*** < 0.001). **e** Images of tumours in nude mice bearing T24 cells treated with normal, normal supplemented with CQ, starvation only and starvation supplemented with CQ. **f** Tumour volume was measured after different treatments in nude mice every 3 days. The arrow indicates the timing of intraperitoneal administration. **g** and **h **Cell Transwell migration and invasion assays were performed in T24 and UM-UC-3 cells with different treatments (n = 3, unpaired Student’s *t* test, P* < 0.05, P** < 0.01, P*** < 0.001)

Transmission electron microscopy and confocal microscopy were used to observe the formation of autophagosomes in T24 cells. In the starvation group, more autophagosomes and more aggregations of LC3B in the cytoplasm were observed (Fig. 2A, B). The expression levels of P62 and LC3-II were detected, which confirmed that autophagic flux was blocked by chloroquine (CQ) in the late stages or 3-methyladenine (3-MA) in the early stage (Fig. 2C, Additional file 1: Fig. S1A). The CCK-8 assay demonstrated that after incubation with HBSS and CQ, the proliferation of T24 cells was significantly inhibited at 48 h and 72 h in contrast to the starvation-only group (Fig. 2D). Subsequently, normal or starvation-incubated T24 cells were subcutaneously inoculated into nude mice at the same time. Routine culture was carried out, and equal amounts of CQ or 0.9% saline were administered intraperitoneally. All starvation group cells grew faster than normal group cells before intraperitoneal injection. After injection with CQ, the tumour growth rate of the starved group was significantly inhibited (Fig. 2E, F). BLCA cell invasion and migration abilities were significantly increased after starvation compared to the control group cells and the cells treated with CQ. However, the CQ-incubated group did not show a variation (Fig. 2G, H).

### Starvation-induced autophagy regulates BLCA glucose metabolic reprogramming by transcriptional overexpression of LDHA

**Fig. 3:**
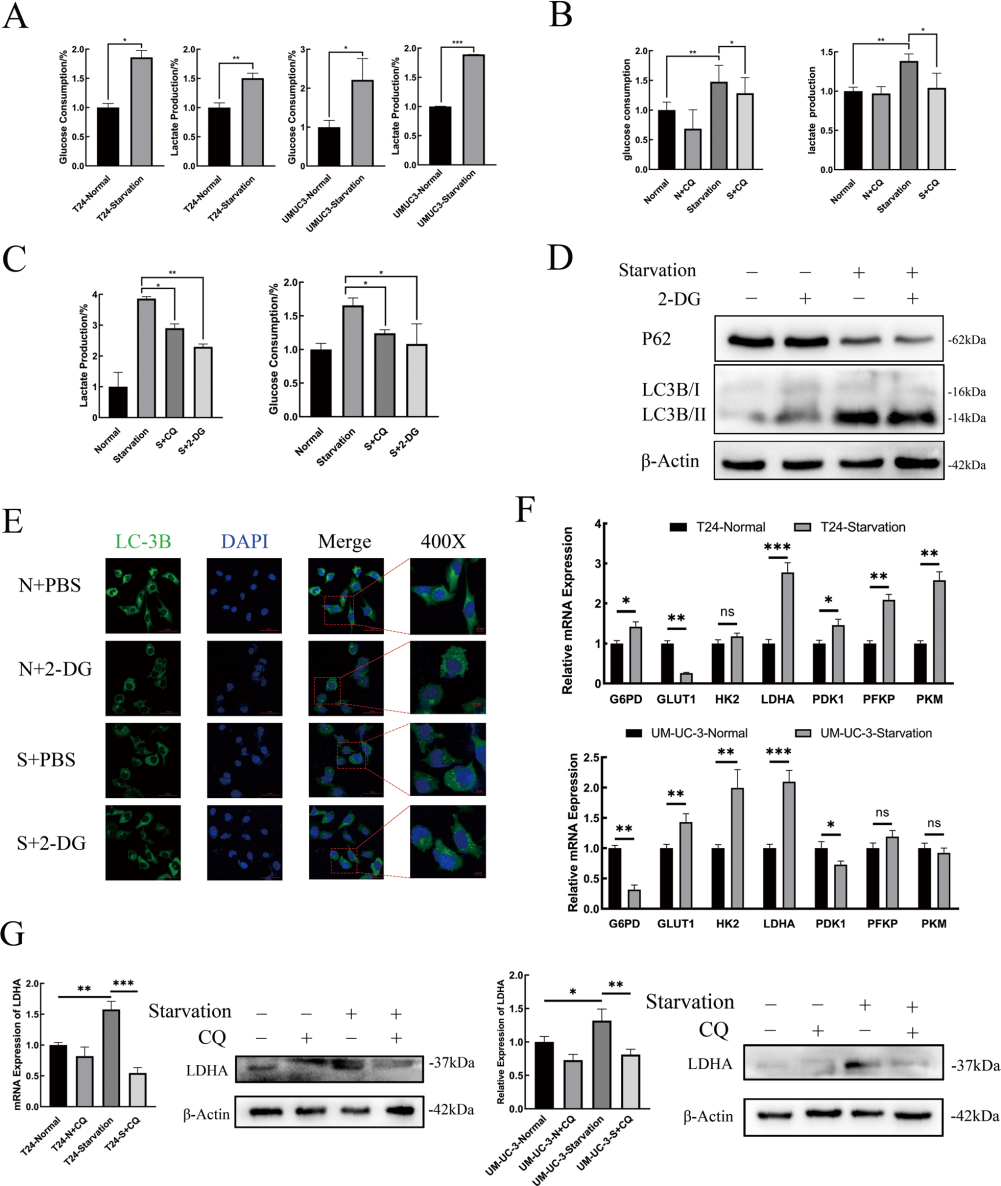
Starvation-Induced Autophagy Regulated BLCA Glucose Metabolic Reprogramming by Transcriptional Overexpression of LDHA. **a** Glucose consumption and lactate production ratios were measured in T24 and UM-UC-3 cells after normal or HBSS treatment, homogenized by cell counting as 10^4^ cells (n = 3, unpaired Student’s *t* test, P ^ns^ > 0.05, P* < 0.05, P** < 0.01, P*** < 0.001). **b** and **c** Glucose consumption and lactate production ratio in T24 were measured in different groups, including normal, normal supplemented with CQ, starvation only, starvation supplemented with CQ or starvation supplemented with 2-DG. **d** Western blotting was used to detect the changes in autophagy levels, reflected by P62 and LC3B-II/I expression in T24 cells under normal, normal supplemented with 2-DG, starvation only and starvation supplemented with 2-DG treatment. **e** Immunofluorescence staining of LC3B was performed in different groups of T24 cells, and the aggregation of LC3B represents the formation of autophagosomes. **f** Real-time qPCR was performed to determine the relative expression levels of key glycolysis enzymes in T24 and UM-UC-3 cells incubated with HBSS or normal control. **g** Real-time qPCR and western blot analysis of the transcriptional and translational expression of LDHA in T24 and UM-UC-3 cells with different treatments. β-Actin was chosen as the loading control

Glucose consumption and lactate production rates were measured. As shown in Fig. 3A, T24 and UM-UC-3 cells both exhibited significantly higher glycolysis levels after starvation (P < 0.05). Then, inhibitors were added to block autophagic flux. The results demonstrated that after treatment with CQ or 3-MA, the glycolysis level of T24 cells was significantly inhibited (Fig. 3B, Additional file 1: Fig. S1B). The exclusive glycolytic inhibitor 2-deoxy-D-glucose (2-DG) was selected to verify the significant increase in glycolytic levels. The glycolysis level of BLCA cells was significantly inhibited after HBSS co-processing with 2-DG (Fig. 3C).

Then, western blot and immunofluorescent staining were used to investigate the effects of inhibiting glycolysis on autophagy. No significant changes were detected in either the degradation of P62 or the decomposition of LC3B after starvation plus 2-DG (Fig. 3D), similar to the aggregation of LC3B in the cytoplasm (Fig. 3E).

The expression profiles of several key glycolysis enzymes were probed at the transcriptional level in the normal and HBSS-treated groups, including G6PD (glucose-6-phosphate dehydrogenase), GLUT1 (glucose transporter type 1), HK2 (hexokinase 2), LDHA, PDK1 (pyruvate dehydrogenase kinase isoenzyme 1), and PFKP. *LDHA* was found to be upregulated most obviously (Fig. 3F). The overexpression of LDHA at the transcriptional and translational levels induced by starvation was verified by RT–qPCR and western blot. After cotreatment with CQ, the expression of LDHA was inhibited significantly with or without starvation (Fig. 3G).

### Starvation-induced metabolic reprogramming promotes the progression of BLCA by LDHA

**Fig. 4:**
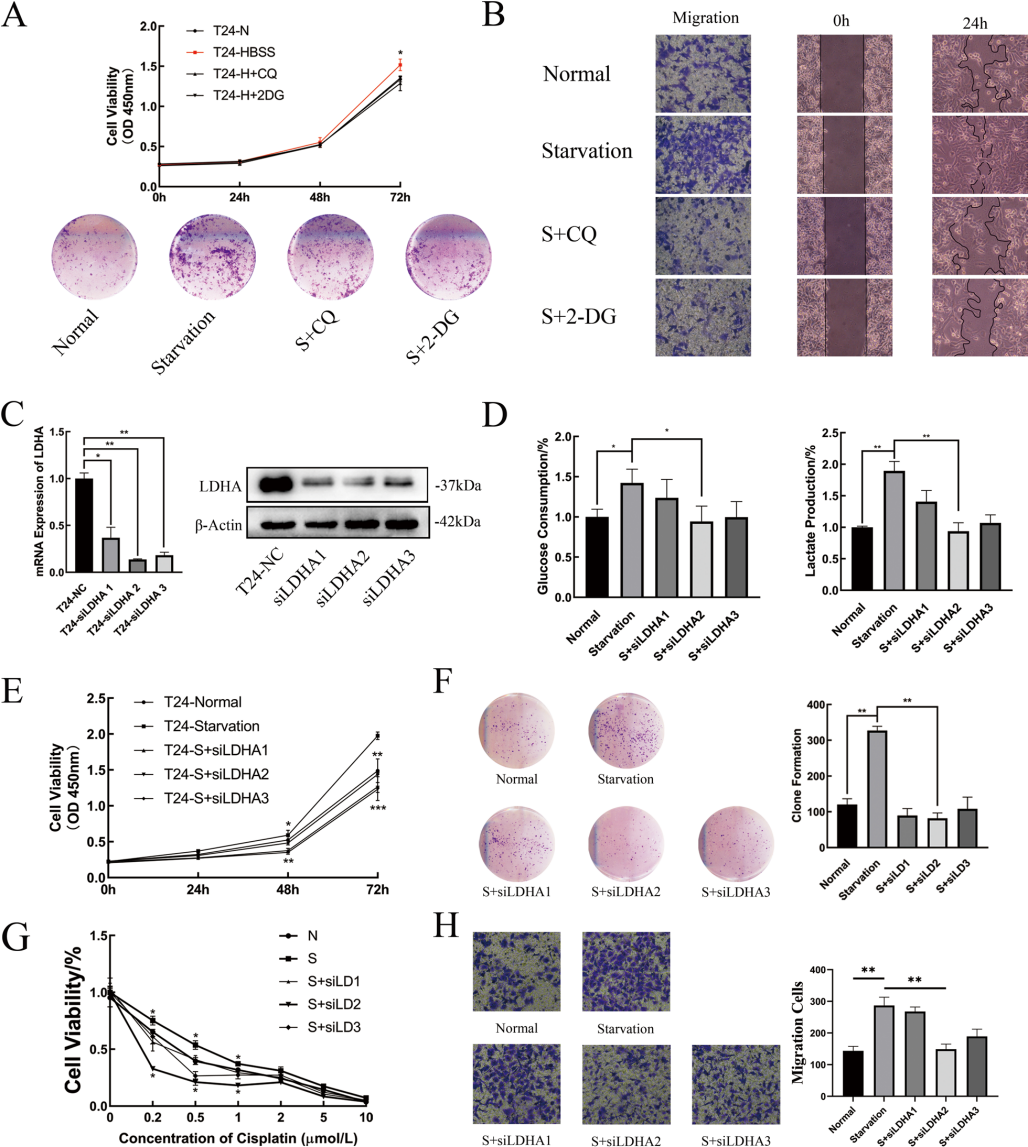
Starvation-Induced Metabolic Reprogramming Promoted the Progression of BLCA by LDHA. **a** Cell proliferation abilities in T24 cells with normal, starvation only, starvation supplemented with CQ and starvation supplemented with 2-DG treatment were assessed by CCK-8 assay and colony forming assay (n = 3, Mann–Whitney test, P* < 0.05). **b** Cell migration abilities in different groups of T24 cells were measured by Transwell and wound healing assays. **c** Real-time qPCR and western blot analysis of the transcription and translation expression levels of LDHA in T24 cells after transfection with siRNAs. β-Actin was chosen as the loading control (n = 3, unpaired Student’s *t* test, P* < 0.05, P** < 0.01). **d** Glucose consumption and the lactate production ratio in T24 cells were measured in different groups, including normal, starvation only, T24 cells transfected with different siRNA sequences then cultured in HBSS and homogenized by cell counting 10^4^ cells. **e** and **f** Cell proliferation abilities in T24 cells in different groups determined by CCK-8 assay and colony-forming assay. **g** The viability of T24 cells was measured after processing with graded concentrations of cisplatin for 48 h, and the cells were divided into different groups. **h** Cell migration abilities in different groups of T24 cells were measured by Transwell assay

With the use of either CQ or 2-DG, BLCA cell starvation-induced proliferation was inhibited in the CCK-8 and clone formation assays (Fig. 4A). When measuring the migration ability, the same trend was observed in the Transwell and wound healing assays (Fig. 4B). These results indicate that starvation-induced autophagy may regulate glucose metabolic reprogramming by affecting the expression of LDHA at the transcriptional level. Small interfering RNAs (siRNAs) targeting LDHA were constructed to evaluate their role in the progression of BLCA cells under starvation conditions. RT–qPCR and western blotting were both used to examine the efficiency of the three siRNA sequences (Fig. 4C, Additional file 1: Fig. S1C).

T24 cells were selected to inhibit the expression of LDHA. Changes in glycolysis levels and their effects on progression in different groups were evaluated. First, silencing LDHA suppressed starvation-induced glycolysis. The second sequence, in particular, exhibited the strongest inhibition of glucose consumption and lactate production (Fig. 4D). Second, the impacts of silencing LDHA on the starvation-induced progression of BLCA cells were examined. The results suggested that starvation-induced BLCA cell progression was suppressed by silencing LDHA (Fig. 4E–H).

### Overexpression of LDHA is closely related to clinical BLCA patients

**Fig. 5:**
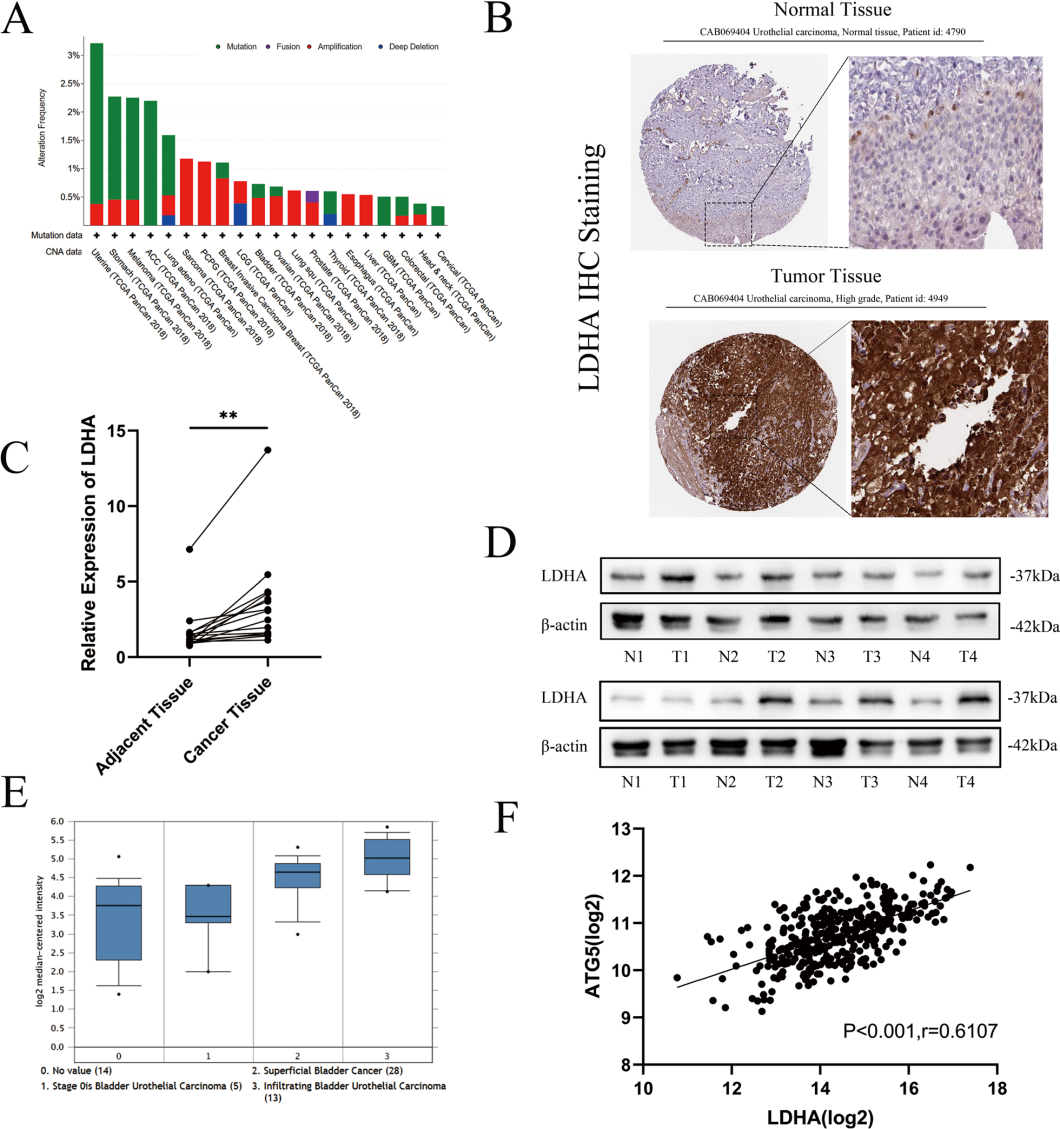
Overexpression of LDHA Was Closely Related to Clinical BLCA Patients. **a** The mutation features and alteration frequency with mutation type of LDHA for TCGA tumours were demonstrated with the cBioPortal tool. **b** Pictures of immunohistochemical staining with CAB069404 from normal bladder or BLCA tissue showing LDHA protein expression in uroepithelial squamous epithelial cells. **c.** Real-time qPCR was used to determine the expression of *LDHA* mRNA in cancer and adjacent tissues (n = 15, paired Student’s *t* test, P** < 0.01). **d** Western blotting was performed to determine the expression of LDHA proteins in cancer and adjacent tissues, which were randomly selected from eight paired clinical samples. **e** Expression of LDHA in 60 BLCA patients with different depths of infiltration sourced from Oncomine. **f** The transcriptional expression relationship between *LDHA* and *ATG5* in 378 BLCA tissues was analysed by Pearson correlation (r = 0.6107, P < 0.001)

Metabolism reprogramming is one of the main hallmarks of cancer cells [18]. The common mutation sites and structures were predicted using cBioPortal. As shown in Fig. 5A, mutations in LDHA were widespread in different cancers, including BLCA. One such mutation in BLCA is amplification. Immunohistochemical images of normal and cancer urothelium tissue were downloaded from the Human Protein Atlas database. Squamous epithelial cells of tumour tissues showed significantly higher LDHA staining than normal tissues (Fig. 5B). RNA was extracted from 15 BLCA patients’ cancer and adjacent tissues. The results showed that *LDHA* was overexpressed in cancer tissues at the transcriptional level (Fig. 5C). Next, eight specimens were randomly sampled from 15 patients to extract protein and detect the expression of LDHA protein (Fig. 5D).

Cohort Dyrskjot Bladder 3, consisting of 60 BLCA patients with different depths of infiltration, demonstrated that LDHA was expressed at a higher level in deeper infiltration tissues (Fig. 5E). Transcriptome data of 378 BLCA patients downloaded from The Cancer Genome Atlas (TCGA) database were analysed, and LDHA mRNA expression correlated positively with ATG5 mRNA (r = 0.61, P < 0.001) (Fig. 5F).

### Starvation regulates LDHA expression through the canonical Wnt/β-catenin pathway

**Fig. 6:**
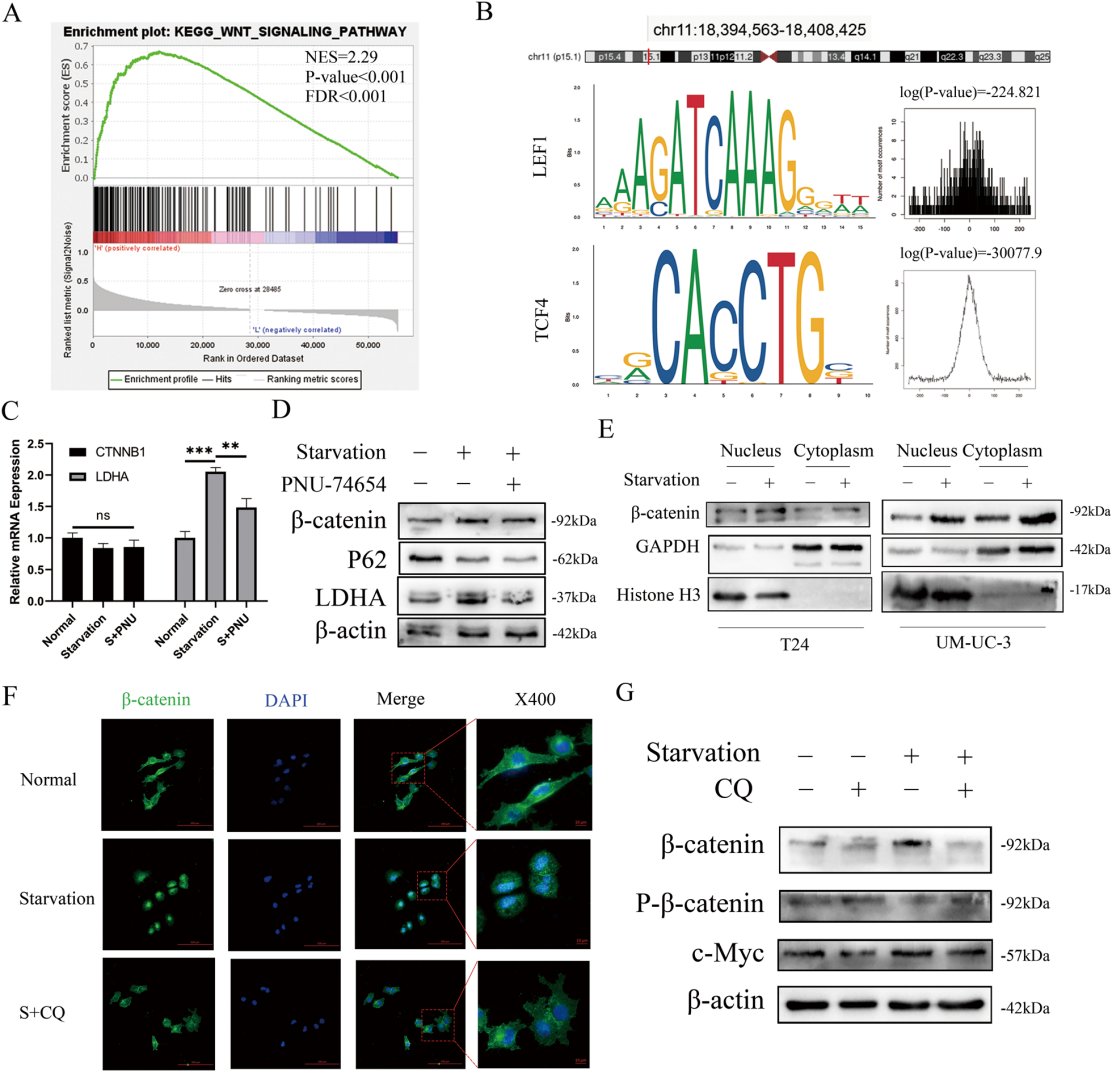
Starvation Regulated the Expression of LDHA through the Canonical Wnt/β-Catenin Pathway. **a** GSEA plots showed that the Wnt/β-catenin pathway was involved in LDHA expression in BLCA. **b** Transcription factor binding sites of LDHA and its location on the chromosome were predicted by USUC and JASPAR. DNA-binding sequences of the transcription factors LEF1 and TCF4 were present in the human LDHA promoter region and were demonstrated with their binding probabilities prediction. **c** Real-time qPCR was used to determine the expression of *LDHA* and *CTNNB1* at the transcriptional level in T24 cells with normal incubation with DMSO, starvation with DMSO and starvation with PNU74654 (n = 3, unpaired *T* test, P^ns^ > 0.05, P* < 0.05, P** < 0.01). **d** Western blots showing TCF4 up- or downstream proteins in T24 cells with different treatments. **E** Expression of β-catenin in the nucleus and cytoplasm of T24 and UM-UC-3 cells treated with or without starvation. Histone H3 was chosen as the nuclear loading control, while GAPDH was used as the cytoplasmic reference. **F** Immunofluorescence staining of β-catenin was performed in T24 cells treated with normal, starvation only or starvation supplemented with CQ, and fluorescence localization was detected by confocal microscopy (scale bar, 8 µm). **g **Western blot analysis presented changes in the expression levels of different proteins in the canonical Wnt/β-catenin pathway in T24 cells under different treatments

Gene set enrichment analysis (GSEA) demonstrated that the expression or function of LDHA was associated with the Wnt signalling pathway (Fig. 6A). With the use of the University of California Santa Cruz Genome Browser and JASPAR, the transcription factor binding site and transcription factors of LDHA were predicted. LEF1 (lymphoid enhancer Factor 1) and TCF4 (T-cell Factor 4) attracted our attention (Fig. 6B) due to their high probabilities of binding to the LDHA transcription factor binding site (TFBS). These factors are members of the TCF/LEF family, and β-catenin specifically binds to and activates the transcription of canonical Wnt pathway target genes [19]. β-Catenin functions by binding with TCF/LEF families and enhancing the expression of Wnt target genes [20]. To verify this prediction, first, PNU-74654 was chosen to selectively block the combination of β-catenin and TCF/LEF [21]. No differences in the mRNA expression of *CTNNB1* were detected among the three groups. However, *LDHA* mRNA expression was significantly enhanced by starvation and inhibited by PNU-74654 (Fig. 6C). Figure 6D shows the overexpression of LDHA by starvation. PNU-74654 did not impair autophagic flux, as reflected by P62. Combined with the RT–qPCR results, we believed that the degradation of β-catenin also reverted when T24 cells were treated with HBSS and PNU-74654. Then, proteins were isolated and extracted from the cytoplasm and nucleus. Glyceraldehyde 3-phosphate dehydrogenase (GAPDH) and Histone H3 were chosen as loading controls. Western blot assays demonstrated that β-catenin levels remained higher after starvation (Fig. 6E). The starvation group showed significant intranuclear high aggregation of β-catenin with the use of immunofluorescence, while the expression of β-catenin in the control group was diffuse and mainly located in the cytoplasm. After autophagy was inhibited by CQ, starvation-induced β-catenin nuclear transposition was blocked (Fig. 6F). Nuclear transfer of β-catenin mainly depends on the reduction of phosphorylation modification. Consequently, the phosphorylation levels of β-catenin and c-Myc were observed. After starvation, the total protein expression levels of β-catenin, c-Myc, and LDHA were increased, and the phosphorylation of β-catenin was decreased (Figs. 3G and 6G). In addition to CTNNB1 expression, there were no differences among these groups (Figure E).

### Starvation regulates the Wnt/β-catenin pathway by ubiquitinating Axin1 and promoting autophagy

**Fig. 7:**
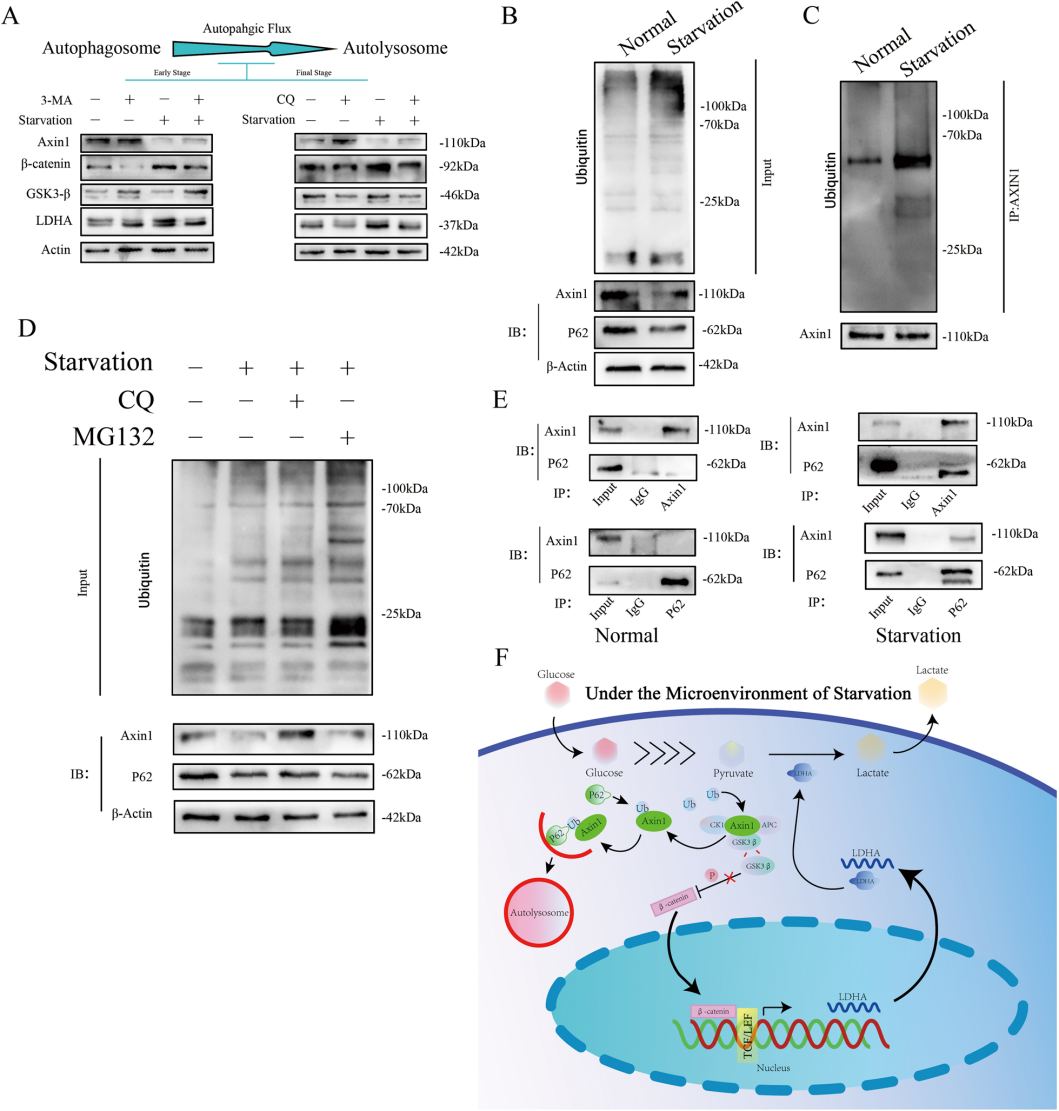
Starvation Regulated the Wnt/β-Catenin Pathway by Ubiquitination of Axin1 and Promotion of Autophagy. **a** Changes in the expression levels of different proteins, including the autophagic flux early-stage inhibitor 3-MA and the final-stage inhibitor CQ, in T24 cells after starvation and treatment with autophagy inhibitors at different stages were detected by Western blot. **b** The amount of ubiquitinated proteins in T24 cells treated with or without HBSS and the expression of Axin1 and P62 determined by western blot. β-Actin was employed as a loading control. **c** Anti-ubiquitin and anti-Axin1 antibodies were chosen to determine the ubiquitination level of Axin1. **d** Western blotting was performed in T24 cells incubated with or without HBSS and treated with DMSO or MG132. **e** Forward and reverse immunoprecipitation assays were chosen to detect the interaction of Axin1 and P62 in T24 cells treated with normal conditions or starvation conditions. **F** A schematic model of starvation-induced enhanced autophagy and glycose metabolic reprogramming realized by LDHA overexpression in BLCA

In the canonical model of Wnt signalling, glycogen synthase kinase 3 (GSK3)-dependent phosphorylation marks β-catenin for degradation at key amino-terminal Ser and Thr residues [22]. Axin1 is considered to be a concentration-limiting factor for the assembly of the β-catenin destruction complex. Degradation of Axin1 enhances the Wnt/β-catenin signalling pathway and increases the expression of downstream genes [23]. We evaluated Axin1 and GSK3-β with the use of the autophagy-lysosomal early- and final-stage inhibitors 3-MA and CQ in the presence and absence of starvation. The results revealed that after starvation, the expression of LDHA and the stabilization of β-catenin were enhanced, which were reversed after autophagic inhibition. Moreover, by blocking autophagic flux at different stages, the degradation of Axin1 could be suspended; nevertheless, no indication was found regarding GSK3-β’s potential involvement with the Wnt/β-catenin signalling activating process during starvation (Fig. 7A).

The ubiquitination levels of both total and Axin1 proteins were detected (Fig. 7B, C). The findings indicated that the ubiquitin modification of Axin1 was increased. Furthermore, the degradation of Axin1 corresponded with that of P62 (Fig. 7B). P62 plays a crucial role in targeting autophagy cargos and autophagosomes for degradation, especially proteins with ubiquitin modification [18]. After inhibition by CQ, the ubiquitin modification on the total protein was demonstrated to be upregulated relative to the control group; no change was observed compared to the starvation-only group (Fig. 7D). In contrast, with increased degradation of Axin1 and P62 after starvation, inhibition of the autophagy lysosomal pathway by CQ also inhibited the degradation of these proteins. Although MG132 blocked the ubiquitin–proteasome system pathway, the degradation of the Axin1 and P62 proteins was not obstructed (Fig. 7D). Taken together, these results indicated that starvation-induced autophagy regulated the degradation of ubiquitin-modified Axin1 and suppressed canonical Wnt/β-catenin signalling by decreasing β-catenin phosphorylation. These results confirm a potential relationship between P62 and Axin1. Forecasted by GeneMANIA, the protein–protein network results showed no correlation between these two proteins (Sfigure F), which was further confirmed by forward and reverse immunoprecipitation in normal T24 cells (Fig. 7E). However, an interaction between P62 and Axin1 in T24 cells was detected after being processed by HBSS. These findings confirm a model of glucose metabolic reprogramming of BLCA cells mediated by starvation-induced autophagy (Fig. 7F).

## Discussion

Previous studies in our group focused on the starvation-induced epithelial‐mesenchymal transition of BLCA [17]. This study explored the process of metabolic reprogramming induced by autophagy and analysed the interaction between ubiquitinated Axin1, the skeleton protein of the APC complex, and P62/SQSTM1, a receptor for autophagy cargos [24], for further degradation by the autophagy–lysosome pathway. Autophagy is a double-edged sword for cancer cells that can result in their survival or death under different circumstances [25]. Hypoxia-induced autophagy could potentially enhance the drug resistance of BLCA to cisplatin [10], and starvation-induced autophagy promotes its metastasis [17]. Several theories have been proposed to explain the progression of cancer induced by aberrant autophagy. Regarding the progression of cancer as a stress-response mechanism is a reasonable explanation [26]. Insufficient angiogenesis usually makes solid tumour cells outgrow their blood supply. This shortage of nutrients and oxygen resulting from the TME generally leads to autophagy. It is important to note that autophagy can promote cancer cell survival under starvation conditions by recycling intracellular components to support metabolism in the absence of extracellular nutrients [27]. Autophagy often regulates the metabolism of cancer cells in three ways: providing obligate substrates for energy needs, regulating mitochondrial metabolism to modulate the supply of energy, and activating key enzyme expression in metabolic pathways [28]. Therefore, it would be reasonable to speculate that autophagy alters metabolic reprogramming by regulating key metabolic enzymes. For instance, autophagy downregulates PtdCho and PtdE biosynthesis in the lipid metabolism of gliomas [2]. Other examples are the decreased expression of arginosuccinate synthase 1 enzyme caused by systemic deletion of ATG7 in circulating arginine [29] and the regulated expression of key enzymes involved in glucose metabolism.

The metabolic patterns of cancer cells are abnormal. They partly acquire energy and components by aerobic glycolysis, and accumulating evidence suggests that glucose metabolic reprogramming is closely related to the malignant progression of cancer cells [2]. Several previous studies have suggested that key enzymes such as PFKP or GLUT1 could promote or inhibit autophagic flux [16, 30], even though most theories argue that autophagy plays a regulatory role in the expression and activity of key enzymes in glycolysis [31, 32]. In this study, after starvation, BLCA cells improved aerobic glycolysis, which could be reversed by inhibition of autophagy. However, when a specific glycolysis inhibitor, 2-DG, was cotreated with starvation, no effects on autophagy were observed. To analyse specific mechanisms, glycolysis-related enzymes in BLCA cells were detected. The results suggested that abnormal overexpression of LDHA was attributed to aberrant glycolysis. LDHA is a rate-limiting enzyme of glycolysis that catalyses the production of lactate, which is relevant to malignant biological behaviours in diverse cancers [33, 34]. LDHA was silenced by siRNA, and the results demonstrated a potential obstacle to BLCA progression. With the use of bioinformatics tools and clinical samples, it was found that the overexpression of LDHA was correlated with not only carcinogenesis but also malignant progression and enhanced autophagy in BLCA. Although our samples were sourced by one institution, which is a limitation with respect to their representativeness, we will continue to investigate whether the overexpression of LDHA in BLCA is prevalent in other BLCA patients. The promoter region of LDHA contains multiple elements that can bind diverse transcription factors [35]. β-Catenin is an important second messenger of canonical Wnt signalling and activates the transcription of Wnt target genes [36]. In the absence of Wnt ligands, β-catenin is confined to the cytoplasm and rapidly sequestered by a destruction complex consisting of Axin1, GSK3, APC, and CK1, which is subsequently marked by GSK3-dependent phosphorylation for further degradation^37^. The Axin1 scaffold determines the efficacy of the destruction complex [38]. After observing the aggregation in the nucleus of β-catenin, reduction of phosphorylated β-catenin, and activation of the canonical Wnt/β-catenin signalling pathway, it was confirmed that starvation-induced autophagy activated LDHA overexpression by destroying the APC complex. Then, two dominant proteins forming the APC complex were detected after blocking autophagic flux at different stages. The SQSTM1/P62 protein plays numerous roles in autophagic flux as a receptor for autophagy cargo. It was also found to mediate the degradation of Axin1 via the autophagy–lysosome pathway. Detailed mechanisms of starvation-induced ubiquitination on Axin1 and combination regions between Axin1 and P62 were not explored in our current study but will be further explored in our subsequent investigations.

## Conclusion

Our study demonstrated that during starvation, the process of autophagy was greatly enhanced, and ubiquitination modification of Axin1 increased and combined with P62 for further autophagy–lysosome degradation. The depolymerized destruction complex liberated β-catenin and promoted its nuclear translocation, binding with LEF1/TCF4 and enhancing the expression of LDHA. Overexpression of LDHA caused glucose metabolic reprogramming and further progressive changes in BLCA cells. Collectively, starvation-induced autophagy promoted glycolysis, metabolic reprogramming and malignant progression in BLCA cells mechanically through the canonical Wnt/β-catenin signalling pathway to enhance the expression of LDHA. Future research will further investigate why ubiquitination modification of Axin1 increases under starvation conditions and how LDHA induces BLCA’s progressive transformation.

## Supplementary Information

**Additional file 1: Figure S1. A.** Western blotting was used to detect the expression of LC3B-I/II and SQSTM1/P62 to verify the efficiency of inhibition of autophagic flux by chloroquine and 3-MA in T24 cells under different treatments. **B.** Glucose consumption and lactate production ratio in T24 were measured in different groups, including normal control, normal supplemented with 3-MA, starvation only, starvation supplemented with CQ and starvation supplemented with 3-MA (n = 3, unpaired students T test, P^*^ < 0.05, P^**^ < 0.01). **c.** Real-time qPCR and western blotting were used to determine the transcription and translation levels of LDHA in UM-UC-3 cells transfected with siRNAs, and β-actin was chosen as the loading control. **d.** The alteration frequency with mutation sites displayed with the highest alteration frequency in the 3D structure of LDHA, marked by red circles. **e.** Real-time qPCR was used to assess the expression of CTNNB1 at the transcriptional level in T24 cells with normal control, normal supplemented with CQ, starvation only and starvation supplemented with CQ. **f.** We analysed a protein–protein interaction network among P62/SQSTM1 and LDHA using GeneMANIA.

## Data Availability

Not applicable.
