# Supplementary figures and images for: Starvation induced autophagy promotes the progression of bladder cancer by LDHA mediated metabolic reprogramming
Source: Cancer Cell Int. 2021 Nov 7;21:597. doi: 10.1186/s12935-021-02303-1 (PMC8573950; doi:10.1186/s12935-021-02303-1)

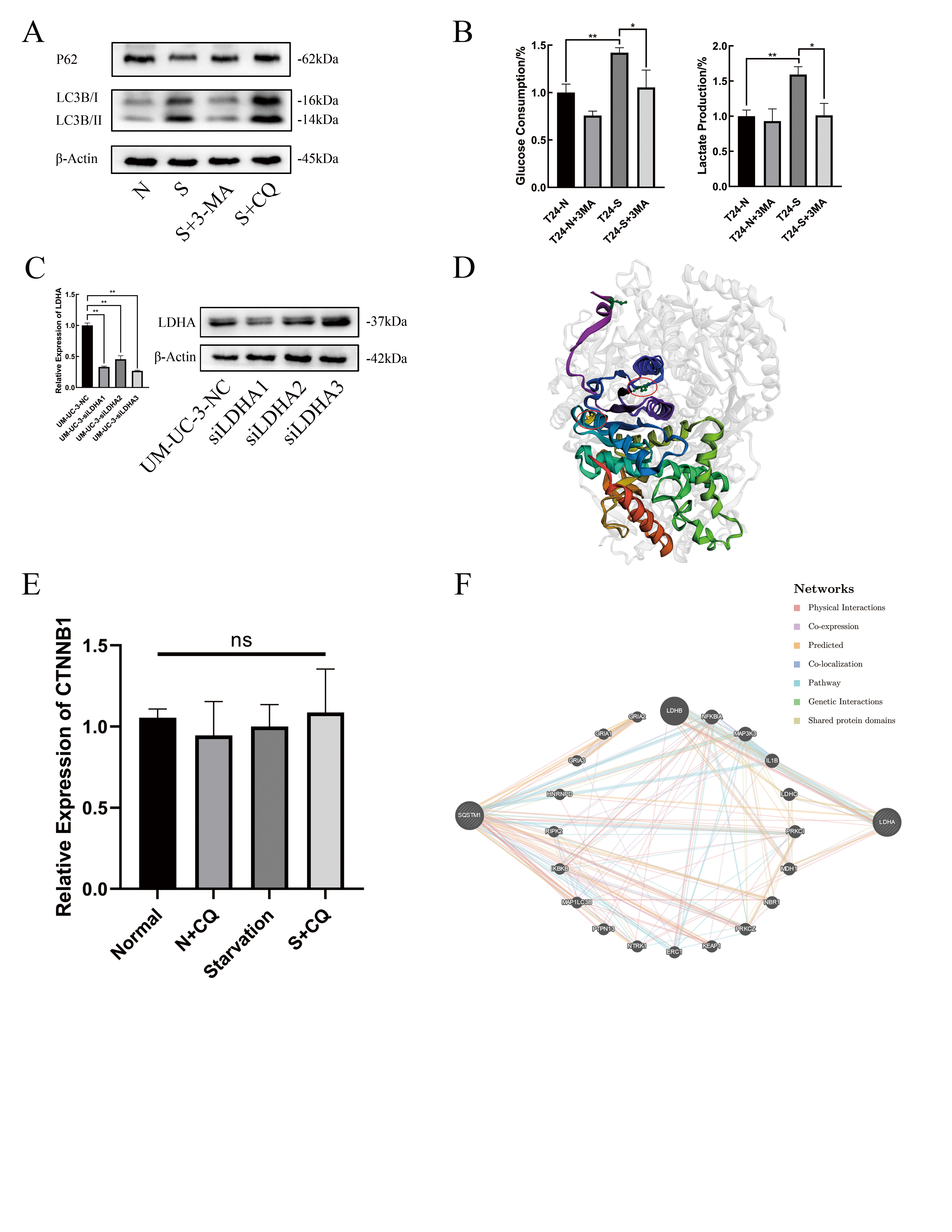

Supplement: Supplementary file 1 — Additional file 1: Figure S1. A. Western blotting was used to detect the expression of LC3B-I/II and SQSTM1/P62 to verify the efficiency of inhibition of autophagic flux by chloroquine and 3-MA in T24 cells under different treatments. B. Glucose consumption and lactate production ratio in T24 were measured in different groups, including normal control, normal supplemented with 3-MA, starvation only, starvation supplemented with CQ and starvation supplemented with 3-MA (n = 3, unpaired students T test, P* < 0.05, P** < 0.01). c. Real-time qPCR and western blotting were used to determine the transcription and translation levels of LDHA in UM-UC-3 cells transfected with siRNAs, and β-actin was chosen as the loading control. d. The alteration frequency with mutation sites displayed with the highest alteration frequency in the 3D structure of LDHA, marked by red circles. e. Real-time qPCR was used to assess the expression of CTNNB1 at the transcriptional level in T24 cells with normal control, normal supplemented with CQ, starvation only and starvation supplemented with CQ. f. We analysed a protein–protein interaction network among P62/SQSTM1 and LDHA using GeneMANIA. [file 12935_2021_2303_MOESM1_ESM.jpg]
